# Meningitis Associated with Simultaneous Infection by Multiple Dengue Virus Serotypes in Children, Brazil

**DOI:** 10.3201/eid2301.160817

**Published:** 2017-01

**Authors:** Paula Eillanny Silva Marinho, Danilo Bretas de Oliveira, Talitah Michel Sanchez Candiani, Ana Paula Correia Crispim, Pedro Paulo Martins Alvarenga, Fabrizia Cristina dos Santos Castro, Jonatas Santos Abrahão, Maria Rios, Roney Santos Coimbra, Erna Geessien Kroon

**Affiliations:** Universidade Federal de Minas Gerais, Minas Gerais, Brazil (P.E.S. Marinho, D.B. de Oliveira, A.P.C. Crispim, J.S. Abrahão, E.G. Kroon);; Hospital Infantil João Paulo II, Minas Gerais (T.M.S. Candiani, P.P.M. Alvarenga. F.C.S. Castro);; Food and Drug Administration, Silver Spring, Maryland, USA (M. Rios);; Fundação Oswaldo Cruz, Minas Gerais (R.S. Coimbra);; Universidade Federal dos Vales do Jequitinhonha e Mucuri, Minas Gerais (D.B. de Oliveira)

**Keywords:** viral meningitis, central nervous system, dengue virus, dengue virus co-infections, dengue virus in children, flavivirus, Brazil, Minas Gerais, viruses

## Abstract

To determine the causes of viral meningitis, we analyzed 22 cerebrospinal fluid samples collected during the 2014–2015 dengue epidemics in Brazil. We identified 3 serotypes of dengue virus (DENV-1, -2, and -3), as well as co-infection with 2 or 3 serotypes. We also detected the Asian II genotype of DENV-2.

Dengue is a disease of high incidence and a major public health problem worldwide (*1*). Approximately 2.5 billion persons live in dengue transmission risk areas, and 50 million dengue virus (DENV) infections occur annually. This disease is endemic in Brazil, with 4 DENV serotypes circulating; >1.6 million clinical cases were reported in 2015 (*2*).

DENV belongs to the family *Flaviviridae*, genus *Flavivirus*, and has 4 serotypes (DENV-1–4). These viruses are usually associated with a systemic and dynamic disease; the clinical conditions range from a nonspecific viral syndrome to severe disease. The most common symptoms are fever, rash, headache, nausea, vomiting, retro-orbital pain, and weakness (*3*). Neurologic manifestations have been increasingly reported; DENV could be considered an emergent etiologic agent of central nervous system (CNS) infection that causes encephalopathy, encephalitis, and meningitis (*1*). DENV infections of the CNS may or may not be associated with the classical systemic manifestations of dengue (*4*).

In dengue-endemic areas, co-circulation of different serotypes has been reported (*5*). Co-infection by different DENV serotypes has already been reported in patients and arthropods, but the effects on the disease and on the virus cycle has not been well established (*6*,*7*). The molecular diversity of DENV serotypes has been linked to different patterns of virulence. DENV-3 genotypes I and III, which circulate in Brazil, demonstrate distinct biological characteristics in mouse models (*8*). In 2014, the number of suspected dengue cases in Brazil was 589,107, with 58,177 in the state of Minas Gerais; in 2015, the number of cases increased to 1,649,008 in Brazil and 189,378 in Minas Gerais, with DENV-1 being the most frequently detected serotype (*2*). We report the detection of DENV-1, -2, and -3 co-infections in the CNS by reverse transcription PCR (RT-PCR) from cerebrospinal fluid (CSF) samples that tested negative for other classic neurotropic pathogens. 

## The Study

During the 2014–15 DENV epidemic in Minas Gerais, 22 CSF samples were collected from children suspected of having viral CNS infection who were hospitalized at the Hospital Infantil João Paulo II, Belo Horizonte, Minas Gerais, a reference children’s hospital for all counties of the state. A presumptive diagnosis of CNS viral infection was given when the CSF of patients with clinical signs and symptoms of CNS infection had normal or slightly altered cytochemical parameters and tested negative for bacterial pathogens (*9*). The protocol for this study was approved by the hospital’s scientific and ethical committee (no. 132/2009), and consent was obtained from parents or accompanying relatives.

The CSF samples tested negative for typical neurotropic viruses such as enteroviruses and human herpesviruses 1, 2, and 3. For DENV detection, RNA isolation and RT-PCR from 140 µL of CSF targeting the NS5 region was performed as described (*10*). The analyses showed that 7 samples (32%) were DENV positive. DENV-1 was detected in 1 sample (14.2% of positive samples); DENV-2 was detected in 3 samples (42.9%); DENV-3 was detected in 1 sample (14.2%); and DENV-4 was not detected in any samples. Co-infection with >1 DENV serotype was found in 2 CSF samples (28.6%); 1 sample was co-infected with DENV-2 and DENV-3 (sample from patient 571), and the other sample was triple infected with DENV-1, -2, and -3 (sample from patient 557) (Table).

**Table:** **I**nformation and laboratory analyses during hospital admission of pediatric patients suspected of having viral CNS infection, Minas Gerais, Brazil*

Patient no.	Age, y/sex	Days hospitalized	Hemogram†				CSF‡				RT-PCR result	Hospital diagnosis	DENV IgM
			Hct	PLT	Leuk		Protein	Glucose	Leuk	PMN			
100	3/F	70	42.1	205	8.4		50	75	30	40	DENV-1	Dengue	+
557	9/F	6	38.1	206	17.4		30	70	28	29	DENV-1/2/3	Dengue	+
571	9/F	3	43.1	243	9.7		24	63	15	86	DENV-2/3	Dengue/viral meningitis	+
572	0.6/M	ND	35.2	365	8		11	54	2	4	DENV-3	Viral meningitis	–
575	0.9/F	5	23.3	522	10.7		97	65	176	1	DENV-2	Viral meningitis	–
577	6	8	39.1	230	9		35	62	42	10	DENV-2	Dengue	+
606	0.4/M	3	35	507	7.1		34	45	5	2	DENV-2	Acute otitis	–

*CNS, central nervous system; CSF, cerebrospinal fluid; DENV, dengue virus; Hct, hematocrit; leuk, leukocytes; ND, not done; PLT, platelets; PMN,  polymorphonuclear neutrophils; +, positive; –, negative. †Units: Hct, %; PLT, x 10^3^/mL; leuk, × 10^3^ cells/mm^3^. ‡These laboratory analyses correspond to the first examination made during the acute phase of CNS infection. Units: protein, g/L; glucose, mmol/L; leuk, × 10^3^ cells/mm^3^; PMN, %.

Retrospective analysis of medical records provided us with information on evolution of the clinical condition of the 7 DENV-positive patients (Table). Patients 575 and 572 received a presumptive diagnosis of meningitis on the basis of clinical signs and symptoms, but they did not show the classical symptoms and signs of dengue fever. Thus, serologic testing for dengue was not requested by clinicians. Patients 100, 557, 571, and 577 were admitted to the hospital with suspected dengue fever. In addition, they demonstrated some neurologic alterations such as seizures; among those patients, only patient 571 showed signs of meningitis. Patient 571 had a predominance of polymorphonuclear neutrophils in the CSF. However, the leukocyte count was <400 cells/mm^3^, and the bacterial culture was negative (*3*).

To confirm DENV as the etiologic agent of CNS infection, we used a 3730 DNA Analyzer (Applied Biosystems, Foster City, CA, USA) to directly sequence the amplified DNA. The nucleotide sequences were aligned and used to construct phylogenetic trees by using the neighbor-joining method (Figure). The Tamura-Nei statistical model implemented in MEGA 6.0 software (Arizona State University, Tempe, AZ, USA) was used with 1,000 bootstrap replicates. These sequences were deposited in GenBank (sequence and accession nos.: 557 CNS DENV-1, KU615569; 557 CNS DENV-2, KU726002; 557 CNS DENV-3, KU948725; 571 CNS DENV-2, KU948727; and 571 CNS DENV-3, KU948726).

**Figure:**
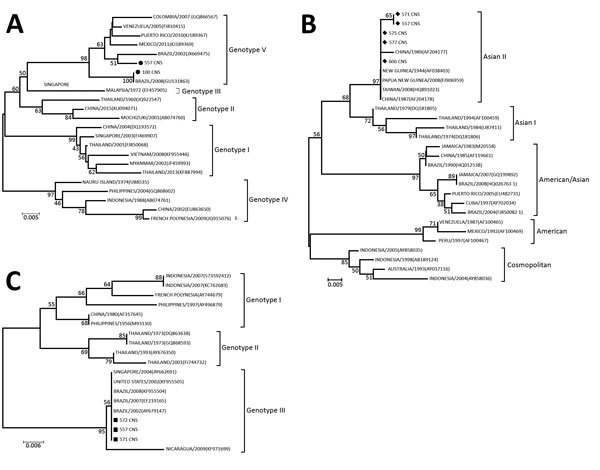
Phylogenetic tree of dengue virus (DENV) isolates obtained from children with meningitis during dengue epidemics, Brazil, 2014–15. A) DENV-1; B) DENV-2; C) DENV-3. The neighbor-joining tree was constructed based on the partial NS5 gene sequences. Sequences from this study (black circles in panel A, diamonds in panel B, squares in panel C) were compared with sequences retrieved from GenBank (accession numbers shown in parentheses). Scale bars indicate nucleotide substitutions per site.

The 3 DENV serotypes found in CSF co-infection have been classified into genotypes, as described (*11*). Phylogenetic analysis of the NS5 gene sequences demonstrated that isolate 557 CNS DENV-1 grouped with isolates of American/African genotype V (Figure, panel A), which has been reported in Southeast Asia (Singapore), South America, and Africa and is the genotype that circulates in Brazil (*12*).

The DENV-2 isolates from samples from patients 557 and 571 grouped with isolates of Asian II, which is a genotype that consists of viruses circulating in China, the Philippines, Sri Lanka, Taiwan, and Vietnam (*13*). The American/Asian genotype has been the only genotype previously identified in Brazil, although some variations have occurred within this genotype (*12*) (Figure, panel B). Singh et al. demonstrated that the Asian II and Cosmopolitan DENV-2 genotypes co-circulated in Nepal, with no differences in their replication rate in mosquito cells (*14*,*15*).

The DENV-3 nucleotide sequences of samples from patients 577 and 571 showed a close phylogenetic relationship with genotype III when compared with the nonstructural-5 gene sequences of other DENV-3 viruses circulating in different parts of the world (Figure, panel C). Previous studies have reported circulation of DENV-3 genotypes I and III in Brazil, and differences in the course of the infection between these 2 genotypes in a mouse model have been described. This study relates DENV-3 genotype III to CNS infection (*8*). DENV serotype co-infection has already been described in the literature from human blood samples and in *Aedes* mosquitoes (*5*–*7*). However, to our knowledge, DENV serotype co-infections have not previously been detected in the CNS.

## Conclusions

DENV co-infection with other flaviviruses has been described, but not in relation to the different DENV serotypes involved in CNS infection. We identified 2 patients with meningitis: 1 was infected with 2 DENV serotypes and the other with 3 DENV serotypes. We also report the circulation of the DENV-2 genotype Asian II in Brazil, where the DENV-2 genotype American/Asian has been the most prevalent genotype since 1990 (*13*). When and where genotype Asian II started to circulate in Brazil is unclear.

Our results also suggest that CNS DENV infection can be preceded by classic dengue fever symptoms or can occur without any classic symptoms. Because the evolution of DENV infection and DENV serotype co-infection into a CNS infection is underreported, attempts to identify the serotypes and genotypes involved in this severe clinical manifestation should be undertaken to clarify the clinical relevance of cases of DENV serotype co-infections.
